# A review of methods and tools to assess the implementation of government policies to create healthy food environments for preventing obesity and diet-related non-communicable diseases

**DOI:** 10.1186/s13012-016-0379-5

**Published:** 2016-02-04

**Authors:** Sirinya Phulkerd, Mark Lawrence, Stefanie Vandevijvere, Gary Sacks, Anthony Worsley, Viroj Tangcharoensathien

**Affiliations:** 1 School of Exercise and Nutrition Sciences, Faculty of Health, Deakin University, Victoria, Australia; 2 Centre for Physical Activity and Nutrition Research, Faculty of Health, Deakin University, Victoria, Australia; 3 Department of Epidemiology and Biostatistics, School of Population Health, Faculty of Medical and Health Sciences, University of Auckland, Auckland, New Zealand; 4 WHO Collaborating Centre for Obesity Prevention, Deakin University, Victoria, Australia; 5 International Health Policy Program, Ministry of Public Health, Nonthaburi, Thailand

**Keywords:** Assessment, Implementation, Healthy food environments, Food policy, Obesity

## Abstract

**Background:**

Policies to create healthy food environments are recognized as critical components of efforts to prevent obesity and diet-related non-communicable diseases. There has not been a systematic review of existing methods and tools used to assess the implementation of these government policies. The purpose of this study was to review methods and tools used for assessing the implementation of government policies to create healthy food environments.

The study conducted a systematic literature search. Multiple databases as well as the grey literature were searched. All study designs and review papers on assessing the implementation of government policies to create healthy food environments were included. A quality assessment of the methods and tools identified from relevant studies was carried out using the following four criteria: comprehensiveness, relevance, generalizability and feasibility. This quality assessment was completed by two independent reviewers.

**Results:**

The review identified 52 studies across different policy areas, levels and settings. Self-administered questionnaires and policy checklists were most commonly applied to assess the extent of policy implementation, whereas semi-structured interviews were most commonly used to evaluate the implementation process. Measures varied widely, with the existence of policy implementation the aspect most commonly assessed. The most frequently identified barriers and facilitators for policy implementation were infrastructure support, resources and stakeholder engagement. The assessment of policy implementation on food environments was usually undertaken in combination with other policy areas, particularly nutrition education and physical activity. Three tools/methods were rated ‘high’ quality and 13 tools/methods received ‘medium’ quality ratings.

**Conclusions:**

Harmonization of the available high-quality methods and tools is needed to ensure that assessment of government policy implementation can be compared across different countries and settings and over time. This will contribute to efforts to increase government accountability for their actions to improve the healthiness of food environments.

**Electronic supplementary material:**

The online version of this article (doi:10.1186/s13012-016-0379-5) contains supplementary material, which is available to authorized users.

## Background

Unhealthy food environments, particularly the greater availability of and access to heavily marketed ultra-processed food products [1], play a significant role in creating unhealthy diets [2, 3] which are one of the major risk factors of obesity and diet-related non-communicable diseases (NCDs) [4].

Food environments have been defined as the collective physical, economic, policy and sociocultural surroundings, opportunities and conditions that influence people’s food and beverage choices and nutritional status [5]. Food environments are complex and are composed of multiple aspects, including food composition, food labelling, food marketing, food retail, food provision, food prices and food in trade and investment agreements [5, 6]. It is well recognized that efforts to improve the healthiness of food environments will need multi-level, multi-actor engagement [7].

Globally, there has been limited implementation of government policies to create healthy food environments [8]. Those policies that have been implemented include nutrition information panels, front-of-pack labelling and regulations on the use of nutrition and health claims on foods, provision of healthy foods and nutrition standards in public institutions and other specific settings, economic tools to address food affordability, restricting unhealthy food advertising to children, improving nutritional quality of the whole food supply, incentives and rules to create a healthy retail and food service environment, and zoning laws and policies to place limits on the density or location of quick serve restaurants or other outlets selling mainly unhealthy foods in communities [9].

In the context of the limited implementation of government policies, there have been recent calls to increase accountability for government action to increase the healthiness of food environments. The assessment and evaluation of policy implementation is increasingly being recognized as a key mechanism for enhancing government accountability [10–13].

High-quality methods and tools are needed to conduct this assessment and evaluation. However, there has not been a systematic review of the quality of existing methods and tools used to assess the implementation of government policies related to food environments.

The objective of this study was to review and assess the quality of existing methods and tools used to assess the government implementation of food environment policies. This will help to inform the choice and harmonization of methods and tools for assessing the implementation of government policies and the implementation process to create healthy food environments for preventing obesity and diet-related NCDs [13]. The harmonization of methods and tools for assessment of policy implementation is considered valuable to compare the extent of policy implementation and barriers/facilitators to policy implementation across countries.

## Methods

We conducted a systematic search of published and grey literature to review methods and tools used to assess governments’ implementation of policies and actions to create healthy food environments for preventing obesity and diet-related NCDs. The grey literature in this review refers to non-academic publications, including publically available documents such as government reports, newsletters, fact sheets, working papers, technical reports, conference proceedings and policy documents. Recognizing the broad extent of existing literature on assessment and evaluation of policy impacts and outcomes, we focused on assessing the quality of the methods used for assessing the extent of policy implementation and the policy implementation process, including barriers and facilitators to policy implementation.

We first performed a search of peer-reviewed literature using the following electronic databases: MEDLINE (1950 to March 2015), Scopus (1960 to March 2015), Cochrane Library (1898 to March 2015) and Web of Science (1964 to March 2015). Then, reference lists of included articles were searched for additional relevant studies. Websites of international health, food and nutrition organizations (i.e. World Health Organization (WHO) including WHO regions, Food and Agriculture Organization of the United Nations, International Food Policy Research Institute, Organisation for Economic Co-operation and Development, Institute of Medicine (USA)) were hand-searched in order to identify additional publications. Websites pertaining to government organizations related to health, food and agriculture in countries mentioned on the aforementioned websites (i.e. UK, Canada, Australia, New Zealand, Norway, Finland, Scotland, South Africa, Ghana, Thailand and the USA) were also consulted for additional documents.

A search strategy was developed for MEDLINE and revised appropriately for the other databases. The key search terms were based on definitions of different aspects of food environments, developed by the International Network for Food and Obesity/NCDs Research, Monitoring and Action Support (INFORMAS) [14]. These key aspects of food environments include food composition, food labelling, food promotion, food prices, food provision, food retail, food production and food trade and investment. These search keywords were used in combination with other groups of keywords which covered the following: ‘monitoring’ and/or ‘evaluation’ or ‘assessment’, ‘government policy’ and/or ‘government action’, and ‘obesity’ and/or ‘NCDs’. Searches through Medical Subject Headings (MeSH) for MEDLINE were conducted to identify other synonyms for the original keywords to be included in the search strategy.

The following search strategy was developed for MEDLINE: (“Policy”[Mesh]) AND (Public OR Government) AND (environment* OR (“Nutritive Value”[Mesh] OR food composition*) OR (“Food Labeling”[Mesh] OR “Food Labeling”[Mesh]) OR (“Marketing”[Mesh] OR food promotion OR food marketing) OR (food tax* OR beverage tax* OR food subsid* OR food pricing) OR (food retail* OR food availability OR zoning* OR outlet density OR outlet proximity) OR (food provision OR food service) OR (food trade* OR food investment OR food production)) AND (“Evaluation Studies as Topic”[Mesh] OR Monitor* OR benchmark*) AND (obes* OR non-communicable disease* OR noncommunicable disease* OR diabetes OR cancer* OR cardiovascular disease* OR coronary heart disease*).

Potentially relevant papers and documents which met the following criteria were selected by screening the titles and abstracts. The criteria for inclusion were that the study had to (1) assess the existence and/or level of implementation of policies and actions, or the implementation process of policies and actions; (2) cover policy aimed at improving the healthiness of food environments for preventing diet-related NCDs, including their risk factors, such as obesity; (3) cover policy developed by governmental bodies and officials; (4) be written in English and published up until March 2015; and (5) specify the tools used. The full texts of relevant articles for which the relevance could not be determined from the abstract alone were also examined. Studies which only focused on government policies and actions directed at the treatment or management of obesity and diet-related NCDs were excluded.

### Quality assessment of methods and tools

The quality and feasibility of methods and tools included in this review were assessed. There are many different sets of criteria for assessing the quality and feasibility of research methods [15–20], but due to the nature of the tools and methods identified in this review (including both quantitative and qualitative methods and highly specific subject matter), no relevant tools were found that could provide a relevant overall assessment of the quality of study tools and methods. This study thus selected the criteria based on a review of the public health and political science literature to determine the assessment criteria most commonly used to assess the quality and feasibility of methods and tools [15–20]. This was supplemented by the authors’ judgement on the applicability of assessment criteria for this study that includes both quantitative and qualitative studies. The following four criteria were considered most relevant to critically assess the quality of the methods and tools used for measuring policy implementation in this context: comprehensiveness, relevance, generalizability and feasibility.

All tools and methods were assessed against these criteria, and the results were combined to form an overall quality rating for each tool/method (refer to Additional file 1 for more details of criteria and standards for quality assessment of the methods used). This quality assessment was completed by two independent reviewers in a two-step process. The first reviewer assessed the quality of all studies, and then, the quality of a 10 % random sample of the reviewed studies was assessed independently by the second reviewer. The 10 % of the study sample size is a common practice for random sampling in many research areas, including literature reviews [21–32]. The two reviewers were in consensus on the quality of all papers in the 10 % sample.

## Results

**Fig. 1:**
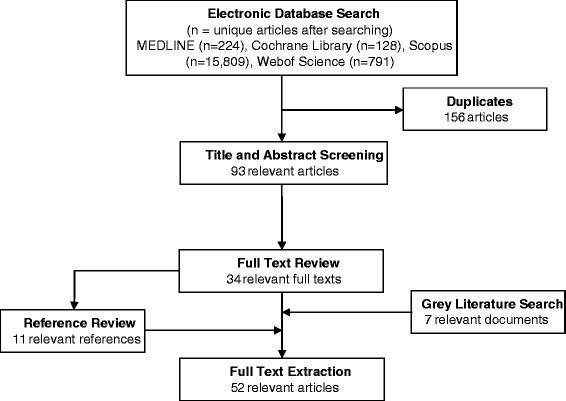
Summary of the literature search process

The extensive search of four electronic databases yielded 16,952 articles. After screening for duplicates, titles and abstracts, and assessment of full texts, there were 34 articles that met the study criteria. In addition, seven published reports from the grey literature and 11 papers identified from the references of already included studies were also included. In total, 52 articles were included in the review (Fig. 1).

Of the identified 52 relevant articles, 24 articles focused on assessing the extent of implementation of food environment policies and actions, 14 articles aimed to evaluate the policy implementation process or barriers/facilitators to policy implementation and 14 articles included both. Forty three of the 52 relevant articles were conducted in high-income countries, two were conducted in low- or middle-income countries and seven were carried out across world regions or at a global level (Table 1).

**Table 1:** Study characteristics of the studies identified in the review

	Number of studies (%)
Study country	
America	
Canada	4 (7.7)
USA	25 (48.1)
Asia Pacific	
Australia	9 (17.3)
New Zealand	2 (3.8)
Europe	
UK	1 (1.9)
Norway	2 (3.8)
Scotland	1 (1.9)
Asia	0 (0.0)
Africa	0 (0.0)
Multiple countries	8 (15.4)
Scope of the study	
Assessing extent of policy implementation	24 (46.2)
Assessing policy implementation process	14 (26.9)
Both assessing the extent of policy implementation and the implementation process	14 (26.9)
Study design	
Quantitative	17 (32.7)
Qualitative	22 (42.3)
Mixed methods	11 (21.2)
Others (guidelines and frameworks)	2 (3.8)

### Assessing the extent of implementation of food environment policies

#### Overview

The literature search yielded 24 relevant studies which specifically focused on assessing the extent of the policy implementation by governments and 14 studies which examined the assessment of extent of the implementation together with the evaluation of the implementation process. Most studies (*n* = 30) were single-country studies, which were conducted in high-income countries, while some (*n* = 8) were multi-country studies, conducted across world regions or at a global level. Both quantitative methods (e.g. self-administered questionnaires) and qualitative methods (e.g. semi-structured interviews, focus group interviews and document review), or a combination of those, were used to assess the policy implementation by governments; however, quantitative methods were more frequently applied. Online supplementary information (Additional file 2) summarizes the identified studies, including implementation measures, key features of the methods and tools used to assess the food environment policy implementation and the overall quality rating of each tool. More detailed results from the quality assessment are provided in Table 2.

**Table 2:** Summary of quality assessment of methods and tools

Methods and tools	Comprehensiveness	Generalizability	Relevance	Feasibility	Overall
Cross-sectional study using qualitative methods with secondary data collected using the WHO global questionnaire tool on assessment of national capacity for NCD prevention and control [4]	L	H	H	H	M
Cross-sectional study using mixed methods with the INFORMAS Healthy Food Environment Policy Index [5, 39, 40]	H	M	H	M	H
Cross-sectional quantitative survey using the WHO Global Nutrition Policy Review questionnaire tool [8]	M	H	H	H	H
Cross-sectional quantitative survey using a policy checklist [38]	L	L	M	M	L
Cross-sectional study using qualitative methods with the EU framework and 8 essential steps proposed by WHO [36]	L	M	M	M	M
Cross-sectional study using qualitative methods with thematic matrix for guiding the interviews [42]	H	M	H	M	H
Cross-sectional quantitative survey using Obesity Action checklist [44]	M	L	H	M	M
Cross-sectional study using qualitative methods with interview protocol [45]	L	L	M	H	L
Cross-sectional quantitative survey using the Audit Form [33]	L	L	L	M	L
Cross-sectional quantitative survey using the Implementation and Measurement Guide of Recommended Community Strategies and Measurements to Prevent Obesity in the USA [46]	M	L	M	H	M
Cross-sectional quantitative survey using scoring policy index system tool [48]	L	L	H	H	L
Cross-sectional quantitative survey using the School Wellness Policies Implementation Questionnaire tool [49]	L	L	L	H	L
Cross-sectional quantitative survey using the Healthy Schools Program tool [50]	L	L	H	H	L
Cross-sectional study using qualitative methods with interview questions based on information from document review [51]	L	L	H	M	L
Cross-sectional quantitative survey using the policy content checklist [52]	L	L	M	H	L
Cross-sectional quantitative survey using the 96-item Wellness School Assessment Tool (WellSAT-96) [47, 54]	L	M	H	H	M
Cohort survey using quantitative methods with adaptive CDC School Health Policies and Programs Study 2000 Questionnaire [37]	L	L	H	L	L
Cross-sectional study using mixed methods with open-ended questions for interviews [55]	L	L	M	M	L
Cross-sectional quantitative survey using the Local Wellness Policy Checklist [58]	L	L	M	H	L
Cross-sectional study using mixed methods with open-ended questions for the focus group [60]	L	M	M	M	M
Cross-sectional study using qualitative methods with the 2004 state semi-annual reports for 21 funded states [61]	L	L	M	H	L
Cross-sectional study using mixed methods with questionnaire tool and list of key questions for discussion [62]	H	L	N/A	M	N/A
Cross-sectional study using mixed methods with tool developed based on Australian core functions of public health as defined by the National Public Health Partnership [63]	L	L	M	M	L
Cross-sectional study using qualitative methods with open-ended questions [53]	L	L	H	M	L
Cross-sectional study using qualitative methods with interview template on policies and regulations on food marketing to children [34]	L	H	H	M	M
Indicators for measuring progress in obesity prevention (method not indicated) [64]	M	L	N/A	H	N/A
Cross-sectional quantitative survey using adapted WHO global questionnaire tool on assessment of the national capacity for NCD prevention and control in 2001 [56]	M	M	M	H	M
Process, output and outcome indicators for of implementation of policies and actions recommended in the WHO Global Strategy on Diet, Physical Activity and Health (method not indicated) [65]	H	H	N/A	M	N/A
Cross-sectional study using qualitative methods with open-ended questions [59]	L	L	N/A	H	L
Cross-sectional study using mixed methods with questionnaire and interview guide and observation form [35]	L	L	H	M	L
Cross-sectional study using qualitative methods with interview guide with broad open-ended questions with probes [43]	L	L	L	H	L
Cross-sectional study using mixed methods with the Report Card on Healthy Food Environments and Nutrition for Children in Canada [41]	M	L	M	M	M
Cross-sectional quantitative survey using Iowa Food System Report Card [66]	M	L	N/A	H	N/A
Cross-sectional quantitative survey using tool developed based on the framework of Lester’s 1994 overview of the Australian food and nutrition system [57]	L	L	M	H	L
Cross-sectional quantitative survey using the Political Commitment and Opportunity Measurement-Rapid Assessment Tool [67]	L	M	M	H	M
Cross-sectional study using qualitative methods with the WHO framework to monitor and evaluate implementation of the Global Strategy on Diet, Physical Activity and Health [71]	M	L	N/A	H	N/A
Cross-sectional quantitative survey using the 55-item State Policy Index [77]	M	M	N/A	H	N/A
Cross-sectional study using qualitative methods with open-ended interview questions [70]	L	L	N/A	H	L
Cross-sectional qualitative methods using interview and focus group guides [68]	L	L	N/A	M	L
Cross-sectional study using qualitative methods with interview guide [72]	L	L	N/A	H	L
Cross-sectional study using qualitative methods with open-ended questions, small prompts, probes and follow-up questions [69]	L	L	H	M	L
Cross-sectional study using qualitative methods with discussion guide [74]	L	L	L	M	L
Cross-sectional study using qualitative methods with interview guide [75]	L	L	L	H	L
Cross-sectional study using qualitative methods with semi-structured questions [80]	M	L	M	H	M
Cross-sectional study using qualitative methods with standard recommended focus group protocols [76]	L	L	N/A	H	L
Cross-sectional study using qualitative methods with semi-structured questions [81]	L	L	L	H	L
Cross-sectional study using qualitative methods with semi-structured questions [79]	H	L	M	H	M
Cross-sectional study using mixed methods with questionnaire tool and semi-structured questions [73]	L	L	H	M	L
Cross-sectional study using qualitative methods with open-ended interview questions [78]	L	L	M	M	L

*N/A* not available

#### Policy areas, levels and settings

A small number of studies (*n* = 9) specifically measured the implementation of food environment policies and actions [5, 33–40]. Most of the studies assessed these policies as part of a range of policies to prevent obesity and NCDs. Many studies (68 %) centred on the implementation of policies addressing food environments in combination with either food and nutrition education or physical activity policy or both [4, 41–65].

The studies encompassed eight common policy domains related to food environments [14] namely food composition (*n* = 11), food labelling (*n* = 11), food promotion (*n* = 17), food prices (*n* = 10), food provision (*n* = 29), food retail (*n* = 12), food production (*n* = 6) and food trade and investment (*n* = 3). Several studies examined multiple domains of food environment policy in one study rather than a single domain only. The implementation of food provision policies appeared to be the most frequently examined (75 %) [5, 8, 33, 35, 37–52, 54, 55, 57–60, 62, 64, 65], followed by food promotion [5, 8, 34, 38–42, 44, 46, 48, 52, 56, 62, 64–66] and food retail [5, 39–42, 44, 56, 57, 62, 64–66].

Several studies (*n* = 20) focused on food environment policies at national or federal level [4, 34–37, 45, 47, 49–52, 54–56, 58–61, 66, 67], followed by state or provincial level [33, 43, 44, 46, 48, 53, 63] and local government level [38, 57]. Some studies (*n* = 9) assessed the policies across different levels, from national or state to local levels [5, 8, 39–42, 62, 64, 65].

Implementation of policies in school settings was the most commonly assessed (*n* = 15) [33, 35, 37, 43, 47–52, 54, 55, 58–60] while some studies focused on policies in various settings such as workplaces, schools, hospitals, childcare centres and communities [5, 8, 38–42, 44, 53, 62, 64, 65]. Some studies did not specify a particular setting.

##### Aspects measured by the study

The identified studies assessed various measures of policy implementation. Thirty studies investigated the existence of policy implementation [4, 5, 8, 33, 34, 36–40, 42, 44–46, 48–56, 58, 60–65]. Some studies (*n* = 15) also investigated the level or degree of policy implementation, but different methods were used to classify the different levels of policy implementation [5, 8, 35, 36, 39–41, 43, 47, 48, 57, 59, 62, 63, 66]. For example, the INFORMAS Healthy Food Environment Policy Index (Food-EPI) categorized the degree of implementation of food environment policies compared to international best practice into five levels (from 1 = less than 20 % implementation to 5 = 80–100 % implementation) [5, 39, 40]. Canada’s Report Card on Healthy Food Environments and Nutrition for Children graded the level of implementation of food environment policies and actions from A through F: grade ‘A’ where the policies and actions were successfully implemented so as to affect a large majority of children and youth and ‘F’ where the policies and actions were implemented so as to affect very few children and youth [41]. The School Wellness Assessment Tool grouped the level of policy implementation into ‘fully in place’, ‘partially in place’, ‘under development’ and ‘not in place’ [47, 54]. Other policy implementation measures examined included implementation coverage (low, medium, high) of the policy or policies in targeted settings [8].

#### Methods and tools used to assess policy implementation

The methods used to assess policy implementation varied across and within studies. Of all the studies, 16 used quantitative, ten used qualitative and ten used mixed methods to assess policy implementation. Two studies reported indicators used only. Most of the methods used were self-administered questionnaires which were specifically designed for multi-domain food environment policies and actions combined with other NCD-related policies. The questionnaires required either a written response, typically with specific response options (e.g. yes/no and rating scale) or a verbal response, typically through telephone communications.

Several studies used one method only, such as self-administered questionnaires among stakeholders [8, 33, 37, 38, 44, 46–50, 52, 56–58, 66, 67], reviews of secondary data and public documents [4, 61] or semi-structured interviews with key stakeholders [43, 45, 59]. The studies which applied mixed methods conducted a quantitative questionnaire survey in combination with document review, interviews or focus groups, or a combination of these [5, 35, 39–41, 54, 55, 60, 62, 63]. Some studies combined different types of qualitative methods, such as document review, stakeholders’ interviews, expert consultation and school observations [34, 36, 42, 51, 53].

In total, 17 quantitative tools and 15 qualitative tools were used for assessing the policy implementation through the use of indicators, items or indexes. Key elements of most of the tools include uses of policy indicators or indexes and numerical scoring system especially numerical rating scale and yes/no formats and involvement of government officials in the studies.

Only three tools used received a ‘high’ quality. They were the INFORMAS Food-EPI [5, 39, 40], the WHO Global Nutrition Policy Review questionnaire tool [8] and thematic matrix for guiding the interviews for an evaluation of the Norwegian Action Plan on Nutrition [42]. Eleven studies were rated as ‘medium’ quality [4, 34, 36, 41, 44, 46, 47, 54, 56, 60, 67], and 18 studies were rated as ‘low’ quality [33, 35, 37, 38, 43, 45, 48–53, 55, 57–59, 61, 63]. Quality ratings could not be completed for four studies due to absence or insufficiency of data on some individual criteria [62, 64–66].

### Evaluating the implementation process of food environment policies

#### Overview

The literature search yielded 14 relevant studies which specifically focused on evaluating the policy implementation process and 14 studies which examined the evaluation of the implementation process, together with the assessment of the extent of the implementation. Out of all the identified studies, 27 single studies were conducted in high-income countries and one multi-country study was performed at a global level with WHO Member States. Nineteen studies applied qualitative methods (e.g. semi-structured interviews, focus groups and document review), four used quantitative methods (e.g. self-administered questionnaires) and five used mixed approaches. Online supplementary information (Additional file 3) summarizes the studies including policy implementation measures, key features of methods and tools used to assess barriers and facilitators to policy implementation, and the overall quality rating of each tool. More detailed results from the quality assessment are provided in Table 2.

#### Policy areas, levels and settings

Almost all studies (*N* = 22) examined the implementation process of multi-domain policies which addressed the promotion of healthy food environments together with other areas related to obesity and NCD prevention. Most often, food environment policies were assessed together with either nutrition education or physical activity policy, or both [42, 43, 45, 47, 49, 52, 53, 55, 59, 60, 62, 68–77].

The studies encompassed seven common domains related to food environments namely food composition (*N* = 5), food labelling (*N* = 6), food promotion (*N* = 7), food prices (*N* = 5), food provision (*N* = 22), food retail (*N* = 6) and food production (*N* = 4). Several studies examined multiple domains of food environment policy in one study rather than a single domain only. Food provision (79 %) was the most common food environment domain examined [33, 35, 42, 43, 45, 47, 49, 52, 55, 59, 60, 62, 68, 70, 72–74, 76–80], followed by food promotion [42, 52, 62, 71, 79–81].

Fourteen studies focused specifically at national or federal-level policy [35, 36, 45, 47, 49, 52, 55, 59, 60, 68, 71, 74, 76, 79], seven focused at state level [33, 53, 73, 77, 78, 80, 81] and five focused at subnational level, i.e. provincial [43], county [70], district [72] and local [69, 75] levels. Two assessed the policies at both national and local/subnational levels [42, 62].

School was the most targeted setting to assess the policy implementation process (61 %) [33, 35, 43, 47, 52, 55, 59, 60, 68, 70–74, 76, 78, 81] while some studies (18 %) focused on policies in various settings in a single study such as workplaces, schools, hospitals, childcare centres and communities [42, 53, 62, 69, 80]. One study examined specifically at community level [45]. Some studies did not specify a particular setting [36, 75, 77, 79, 81].

##### Aspects measured by the study

The identified studies mainly investigated the factors (barriers and facilitators) impacting policy implementation. Out of 28 studies, 24 focused on the investigation of barriers and facilitators of policy implementation while four focussed on specific issues or problems, such as infrastructure and resource support, stakeholder engagement, and monitoring and evaluation mechanism for implementation [42, 53, 71, 77]. Key issues which were frequently observed as barriers or facilitators of policy implementation were support for infrastructure and/or resources such as financial and human resources [33, 35, 36, 42, 47, 49, 53, 55, 59, 60, 68–72, 74, 75, 77], stakeholders’ engagement in the policy implementation process and partnerships [35, 36, 43, 47, 59, 60, 68–71, 74, 77, 80], monitoring and evaluation mechanisms for implementation and enforcement issues (such as stakeholders’ resistance and negotiation with private sectors) [36, 49, 55, 59, 60, 68, 70, 71, 76, 77, 80, 81], coordination mechanisms and leadership and implementation governance [35, 36, 42, 47, 49, 70, 75, 76, 79, 80], role of implementers [35, 72, 75, 76, 79], and policy communications among stakeholders [42, 70, 72]. Other influential factors identified include organizational capacity [35, 55], governance [42] and leadership [60, 70].

#### Methods and tools used to evaluate the implementation process

Of all the studies, 19 studies were conducted using qualitative methods, while four studies used quantitative and five used mixed methods. Semi-structured interviews were most commonly used with a list of open-ended questions to facilitate and guide the interview. Most of the tools were originally developed for use in particular countries.

In several cases, one or more types of qualitative methods were used in one single study. In-depth interviews were most commonly used as primary sources of data [33, 42, 43, 45, 53, 59, 68–70, 72–75, 78–81]. Either policy implementers or both policy implementers and other relevant stakeholders were often recruited as informants for in-depth and focus group interviews. Other qualitative methods used include document review, field observation and expert review [35, 36, 53, 62, 71, 78, 79]. Some studies used either a quantitative survey [47, 49, 52, 77] or mixed methods [35, 55, 60, 62, 73] for evaluation.

Twenty-one qualitative tools and eight quantitative tools were reported for evaluating the policy implementation process. Among the qualitative tools used were interview guides, which varied from highly to loosely structured. In some cases, the tools were adapted from existing tools. For example, McDonnell et al. (2006) used standard recommended focus group protocols developed by Krueger and Casey [82]. In several cases, the studies developed their own tools such as a thematic matrix [42], interview and focus group guides [35, 68, 72, 74, 75] and lists of open-ended questions or issues to be explored [33, 43, 53, 55, 59, 60, 62, 69, 70, 73, 78–81]. Among the tools used, seven qualitative tools were presented data in a form of narrative report while three quantitative tools were based on numerical scores with different forms of data presentation, i.e. yes/no [47, 49] and scales from 0 to 5 [77].

Only one tool for assessing the policy implementation process was rated ‘high’ quality, i.e. the thematic matrix for guiding the interviews for an evaluation of the Norwegian Action Plan on Nutrition [42]. Five studies were rated ‘medium’ quality [36, 47, 60, 79, 80], and 19 studies were rated ‘low’ quality [33, 35, 43, 45, 49, 52, 53, 55, 59, 68–70, 72–76, 78, 81]. Three studies provided insufficient information for the assessment [62, 71, 77].

## Discussion

This review identified 52 relevant studies across different policy areas, levels and settings, including 49 tools/methods used for assessing the implementation of government policies to create healthy food environments. The quality of these tools/methods varied widely, with only three tools/methods rated as high quality according to the detailed assessment criteria.

There were some broad similarities in the assessed aspects measured by the study and the methods and tools used. It is clear that policy implementation by governments has been measured in varying levels of detail, such as the existence or absence of policy implementation, level/degree of policy implementation and implementation coverage. Studies evaluating policy implementation processes mainly sought information about barriers and facilitators of policy implementation, particularly infrastructure support and resources, stakeholder engagement, leadership, and available monitoring and evaluation systems, which were the most commonly identified factors which impacted the policy implementation process.

There are no common standard methods and tools used to measure the policy implementation or to assess the policy implementation process. This may be due to the differing contexts and the needs or interests of assessors using these methods. The three tools that were rated as high quality (i.e. the INFORMAS Food-EPI, WHO Global Nutrition Policy Review questionnaire tool, and thematic matrix for guiding the interviews for an evaluation of the Norwegian Action Plan on Nutrition) could provide starting points for researchers and policymakers to identify appropriate methods for use in national and local assessment and evaluation of food environment policy implementation. However, there may be scope to include aspects of other tools as part of assessment methods, depending on context-specific requirements and the particular focus required. For example, the Report Card on Healthy Food Environments and Nutrition for Children in Canada included, combined or adapted indicators of several tools used for measuring progress in creating healthy food environments for obesity prevention to fit its purpose and scope and Canadian context [41].

Consideration should be given to harmonization of the use of methods and tools in this area. While it will always be important to apply tools and methods that are appropriate to the specific context in which they are to be implemented, the use of similar tools in different contexts will allow comparisons across countries and settings and over time. This will also facilitate effective benchmarking of performance which can help contribute to increasing accountability of governments for their actions to improve the healthiness of food environments.

The global impetus to assess policies for changing food environments is relatively new, and the development of appropriate tools for assessing implementation progress in this area is relatively under-developed. In contrast, in other public health policy areas such as tobacco, alcohol and breast milk, tools are relatively more advanced and have been used for assessing changes over time in a range of countries [83–88]. Examples include approaches to measuring breastfeeding policy implementation including the implementation of the international code of marketing of breast milk substitutes by WHO [86], International Baby Food Action Network (IBFAN) [84] and UNICEF [89] and tracking the progress of the implementation of policies and actions in alcohol and tobacco control by WHO [83, 87, 88]. These approaches share commonalities in terms of types of methods used for assessing policy implementation and provide useful means for the development of healthy food environments. Ninety-two countries, for example, have implemented the World Breastfeeding Trends Initiative (WBTi) tool, developed by IBFAN Asia, to track and monitor status and benchmark the progress of implementation of the Global Strategy for Infant and Young Child Feeding [84]. This includes assessment of the strengths and weaknesses of their related policies and programmes. The assessment is conducted every 3–5 years, and the findings and recommendations are actively fed back to policymakers in each country.

The main strength of this study is that it is a comprehensive review based on a thorough and systematic search of the literature for policy assessment and evaluation. To our knowledge, it is the first time such a review has been conducted. The study rated the quality of each tool, and the methods used to conduct the quality assessment could be applied elsewhere. However, this study has several limitations. Firstly, the search was restricted to English-language publications. This may have resulted in the exclusion of important non-English publications. Moreover, studies assessing policy implementation were predominantly from high-income countries rather than low- or middle-income countries. This may be due to literature search being limited to peer-reviewed studies or grey publications published in English only. It may have missed some relevant documents published in languages other than English, especially documents from countries where English is not the main language. Furthermore, the studies identified were conducted in different contexts with different focuses, so they may be difficult to compare. The degree to which an approach used in one context is applicable to other contexts is uncertain. However, our findings are consistent with one recent paper identifying that there is little monitoring for accountability globally in this area [13].

## Conclusion

Although there is a growing concern about the impact of unhealthy food environments on the prevalence and severity of obesity and diet-related NCDs globally and nationally, and some governments have implemented policies to improve the healthiness of food environments, a relatively small proportion of the implementation of these policies and actions is being assessed and evaluated. This review investigated methods and tools used to assess and evaluate the implementation of government policies to create healthy food environments for preventing obesity and diet-related NCDs. It provides a shortlist of high-quality tools and methods for assessing the implementation of such policies. Harmonization of the use of these high-quality methods and tools is needed to ensure that assessment of government policy implementation can be compared across different countries and settings and over time. The findings from the review are timely in that they provide insights for informing policy implementation and strengthening accountability mechanisms in the context of the increasing prevalence of obesity and diet-related NCDs in low-, middle- and high-income countries.

## Additional files

Additional file 1: **Criteria and standards for quality assessment.** (DOC 85 kb)

Additional file 2: **Summary of identified studies assessing the extent of policy implementation.** (DOC 160 kb)

Additional file 3: **Summary of identified studies evaluating policy implementation process.** (DOC 136 kb)

## Abbreviations

Food-EPI: INFORMAS Healthy Food Environment Policy Index
IBFAN: International Baby Food Action Network
INFORMAS: International Network for Food and Obesity/NCDs Research, Monitoring and Action Support
NCDs: non-communicable diseases
WBTi: World Breastfeeding Trends Initiative
WHO: World Health Organization
