# Supplementary material for: A review of methods and tools to assess the implementation of government policies to create healthy food environments for preventing obesity and diet-related non-communicable diseases
Source: Implement Sci. 2016 Feb 4;11:15. doi: 10.1186/s13012-016-0379-5 (PMC4743239; doi:10.1186/s13012-016-0379-5)
Supplement: Supplementary file 1 — Criteria and standards for quality assessment. (DOC 85 kb) [file 13012_2016_379_MOESM1_ESM.doc]

**Additional file 1:** Criteria and standards for quality assessment of methods and tools

| **Criteria for assessment** | **Standards** |
| --- | --- |
| Comprehensiveness | Refers to the ability of the methods and tools to adequately capture core aspects of the study topic, which are food environments in this case. The proportion of key aspects of food environments (as identified by INFORMAS (14)) captured was evaluated as follows:  ‘Low’ comprehensiveness means only 1-2 aspects (food composition, food labelling, food prices, food provision, food promotion, food retail, food production, and food trade and investment) were included.  ‘Medium’ comprehensiveness means 3-5 aspects of food environments were included.  ‘High’ comprehensiveness means > 6 aspects of food environments were included. |
| Generalisability | Refers to the degree to which the results of the study are generalisable to other groups of people or contexts. The methods and tools used in the studies can be assessed as follows:  ‘Low’ if the methods and tools are mostly country-specific.  ‘Medium’ if they are applicable in other countries/contexts.  ‘High’ if they are applicable globally. |
| Relevance | Refers to the degree to which the results of the study are relevant and accurate to the people and conditions assessed by the studies. The assessment takes into account the following aspects: at least 50 percent response rate from participants; measurement tools are pre-tested; the sample is representative of the target population; and reported data are verified by using another source of data (e.g. secondary data and direct observation). The process for assessing relevance can be described in detail by the following steps:  Step 1 Assess all four individual sub-criteria (at least 50% response rate, representative sample, pre-tested tool, verified reported data) using Yes/No rating.  Step 2 Make the overall assessment as follows:  ‘Low’ if the study meets 1 of 4 sub-criteria or does not meet any sub-criteria  ‘Medium’ if a study meets 2 of 4 sub-criteria  ‘High’ if a study meets at least 3 of 4 sub-criteria |
| Feasibility | Refers to the ease and practicality of applying the methods and tools. The following aspects should be taken into account for the assessment: easy to administer (e.g. low human resource and technical skills required), and interpretable (being supplemented by detailed instruction or guides for using the methods and tools and interpreting results).  The feasibility was assessed based on the authors’ judgement by comparing the relative feasibility across these studies. Each study was rated as ‘high’, ‘medium’ or ‘low’ feasibility. |
| **Overall assessment** | The overall quality of the methods and tools for each study was rated as ‘Low’, ‘Medium’ or ‘High’ based on the collective assessment for the four individual assessment criteria (each criteria had the same weighting) as follows:  Low: Two or more Low ratings for any of the assessment criteria.  Medium: Three High plus one Low rating for any of the assessment criteria; four Medium ratings; two High plus one Medium plus one Low rating for any of the assessment criteria; one High plus three Medium ratings for any of the assessment criteria; three Medium plus one Low rating for any of the assessment criteria; or one High plus two Medium and one Low rating for any of the assessment criteria.  High: Four High ratings; three High plus one Medium rating for any of the assessment criteria; or two High plus two Medium ratings for any of the assessment criteria.  Those studies which provided insufficient information to assess against any individual criteria were rated as not applicable (N/A) for the overall quality assessment of methods and tools. |
