# Supplementary material for: A review of methods and tools to assess the implementation of government policies to create healthy food environments for preventing obesity and diet-related non-communicable diseases
Source: Implement Sci. 2016 Feb 4;11:15. doi: 10.1186/s13012-016-0379-5 (PMC4743239; doi:10.1186/s13012-016-0379-5)
Supplement: Supplementary file 2 — Summary of identified studies assessing the extent of policy implementation. (DOC 160 kb) [file 13012_2016_379_MOESM2_ESM.doc]

**Additional file 2: Summary of identified studies assessing the extent of** the government implementation of food environment policies and actions for preventing obesity and diet-related NCDs

| **Author** | **Study country** | **Objective of the study** | **Policy levels and settings** | **Policy areas** | **Aspects measured by the study** | **Design and methods** | **Tools**  **[scales used]** | **Overall quality of method/tool** |
| --- | --- | --- | --- | --- | --- | --- | --- | --- |
| WHO (2011) [4] | Global | To assess the current global status of  NCDs including current state of progress countries are making to address these diseases in terms of  policies and plans, infrastructure, surveillance and population-wide and individual interventions | National level  (detail on setting not given) | NCDs and risk factors such as tobacco, harmful use of alcohol, breastfeeding, physical activity and unhealthy diets  (no specific details on food environments given) | The existence of implementation of policy of WHO Member States to NCDs and risk factors against recommendations of the Global Strategy for the Prevention and  Control of NCDs | Qualitative method:  review of data from self-administered survey questionnaires | The WHO global questionnaire tool on assessment of national capacity for NCD prevention and control  [Yes vs No per item/indicator and comparison to baseline] | Medium |
| Swinburn et al. (2013) [5] | Global | To assess government policies and actions for creating healthy  food environments | National and subnational level, various settings | Food composition, food labelling, food prices, food provision, food promotion, food retail, food production, and food trade and investment | The existence and level of implementation of food environment policies | Mixed methods:  cross-sectional study using document review, expert consultations and self-administered questionnaires with non-government stakeholders | The INFORMAS Healthy Food Environment Policy Index  [0-5 per indicator where 0=less than 20% implemented compared to international best practice, and 5=80-100% implementation] | High |
| WHO (2013) [8] | Global | To assess progress of implementation of existing national policies and institutional environments related to nutrition | National and sub-national levels, various settings | Nutrition-related NCDs and malnutrition (including food labelling, food promotion, food composition, food provision and food prices) | - The existence and level of implementation of nutrition policies and programmes  - Implementation coverage | Quantitative method:  cross-sectional survey using self-administered questionnaires with government officials of the WHO Member States | The WHO Global Nutrition Policy Review questionnaire tool [Yes vs No per indicator and comparison to baseline] | High |
| Ardzejewska et al. (2013) [33] | Australia | To investigate the barriers and facilitators to, and the extent of the implementation of, the New South Wales (Australia) ‘Healthy School Canteen Strategy’ | State level, school setting | Food provision | The existence of implementation of the New South Wales ‘Healthy School Canteen Strategy’ | Quantitative method:  cross-sectional survey using questionnaires with schools | The Audit Form  [number of actions implemented across different schools] | Low |
| Hawkes et al. (2011) [34] | Global | To describe the global regulatory environment around food marketing to children in 2009 and to identify changes in this environment since 2006 | National level  (detail on setting not given) | Food marketing to children | The existence of government policies and actions on food marketing to children | Qualitative methods:  cross-sectional study using document review and in-depth interviews with government and non-government stakeholders | Interview template on policies and regulations on food marketing to children  [proportion of countries with actions on food marketing (%)] | Medium |
| Holthe et al. (2011) [35] | Norway | To examine how schools implemented the national guidelines for healthy school meals and the extent to which the degree of implementation was related to the organizational capacity of schools | National level, school setting | Food provision | The level of implementation of Norwegian national guidelines for healthy school meals | Mixed methods:  cross-sectional study using document review, self-administered questionnaires, focus group interviews with teachers and students, and school observations | Interview guide and observation form  [narrative report]  (tool not specified for questionnaire)  [Yes, Partial, No per element] | Low |
| Rodriguez-Fernandez et al. (2014) [36] | 53 WHO European Member States | To assess current salty reduction policies in countries of the WHO European Region against the backdrop of varying levels of human development adjusted for income, education and health (longevity) inequalities | National level  (details on settings not given) | Food composition and food labelling | The existence and level of implementation of salt reduction policy including infrastructure supports (i.e. industry involvement and monitoring and evaluation mechanism) | Qualitative method:  cross-sectional study using document review and electronic communication with the WHO Nutrition Counterparts in the European Member States | The EU framework and eight essential steps proposed by WHO  [3 levels per indicator where level 1=fully implemented, level2=‘partially implemented/planned, and level3=non-existent] | Medium |
| Seo (2009) [37] | USA  (Indiana) | To investigate the impact of the law in terms  of school food policies and food preparation practices using a prospective design | Federal level, school setting | Food provision | The existence of implementation of school food policies and food preparation practices | Quantitative method:  cohort study using self-administered questionnaires with school principals or food service directors in 2006 and 2007 | Adaptive CDC School Health Policies and Programs Study 2000 Questionnaire [Yes vs No per item and comparison to baseline] | Low |
| Silberfarb. (2014) [38] | USA  (Minnesota) | To assess multicity implementation of  healthful food access policy, systems, and  environmental changes | Local level, various settings | Food provision and food promotion | The existence of implementation of policies including infrastructure supports (i.e. monitoring mechanism) | Quantitative method:  cross-sectional survey using self-administered questionnaires with multiple city officials | A policy checklist  [Yes vs No per indicator] | Low |
| Vandevijvere et al. (2014) [39] | New Zealand | To describes the design and methods of comprehensive national survey on the healthiness of food  environments and the public and private sector policies influencing them | National and subnational level, various settings | Food composition, food labelling, food prices, food provision, food promotion, food retail, food production, and food trade and investment | The existence and level of implementation of policies | Mixed methods:  cross-sectional study using document review, expert consultations and self-administered questionnaires with non-government stakeholders | The INFORMAS Healthy Food Environment Policy Index  [0-5 per indicator where 0=less than 20% implemented compared to international best practice, and 5=80-100% implementation] | High |
| Vandevijvere et al. (2015) [40] | New Zealand | To pilot test the INFORMAS Healthy Food Environment Policy Index  (Food-EPI), and  revise the tool and process | National and subnational level, various settings | Food composition, food labelling, food prices, food provision, food promotion, food retail, food production, and food trade and investment | The existence and level of implementation of policies | Mixed methods:  cross-sectional study using document review, expert consultations and self-administered questionnaires with non-government stakeholders | Food-EPI tool  [0-5 per indicator where 0=less than 20% implemented compared to international best practice, and 5=80-100% implementation] | High |
| Olstad et al. (2014) [41] | Canada | To assess how current environments and policies in Canada support or create barriers to improving children's dietary behaviours and body weights | National, provincial/local and organization levels, various settings | Food and nutrition education, and breast-milk feeding  (including food composition, food labelling, food provision, food promotion and food retails) | The level of implementation of food environment and nutrition policies and actions targeting children | Mixed methods:  cross-sectional study using document review, expert consultations and self-administered questionnaires with researchers, members of the Active Healthy Kids Canada Research Work Group, and policymakers and practitioners | The Report Card on Healthy Food Environments and Nutrition for Children in Canada  [Grade A to F where A=being implemented so as to affect a large majority of children and youth, and F=being implemented so as to affect very few children and youth] | Medium |
| WHO/Europe (2013) [42] | Norway | To evaluate the Norwegian Action Plan on Nutrition 2007–2011 | National and local levels, various settings | Food and nutrition, physical activity and education (including food composition, food provision, food retail, food labelling, food prices and food promotion) | The existence of implementation of the Norwegian Action Plan on Nutrition 2007-2011 | Qualitative methods:  cross-sectional study using document review and in-depth interviews with policy makers and government and non-government stakeholders | Thematic matrix for guiding the interviews  [narrative report] | Medium |
| Mâsse et al. (2013) [43] | Canada | To explore the factors which impeded or  facilitated the implementation of publicly mandated school-based physical activity and nutrition guidelines in the  province of British Columbia (BC), Canada | Provincial level, school setting | Food and nutrition, physical activity and education (including food provision) | The level of implementation of publicly mandated physical activity and food and beverage sales guidelines | Qualitative method:  cross-sectional study using semi-structured interviews with school principals and key teachers/school informants | Interview guide with broad open-ended questions with probes  [narrative report] | Low |
| Martin et al. (2013) [44] | Australia | To develop a benchmarking tool for government action on obesity prevention, implement it across Australian jurisdictions and to publicly award the best and worst performers | State level, various settings | Food and nutrition, physical activity and education (including food marketing, food price and affordability, food retail and food provision) | The existence of implementation of policies and actions on obesity prevention | Quantitative method:  cross-sectional survey using self-administered questionnaires with key non-government informants from each state or territory | The Obesity Action checklist [0-10 per indicator where 0=non-existent action and 10=fully implemented] | Medium |
| Barnidge et al. (2013) [45] | USA | To (1) identify types of environmental and policy interventions being implemented in rural communities to promote physical activity or healthy eating, (2) identify barriers to the implementation  of environmental or policy interventions, and (3) identify strategies rural communities have employed to overcome these barriers | Federal level, community setting | Food and nutrition, physical activity and education  (including food provision and food production) | The existence of implementation of environmental or policy interventions to promote physical activity and/or healthy eating in rural communities | Qualitative method:  cross-sectional study using in-depth interviews with public health professionals from non-profit or local governments | Interview protocol  [number of actions implemented across different communities] | Low |
| Anderson et al. (2013) [46] | USA  (New Hampshire) | To assess the extent to which MP-recommended policies and assets already exist in New Hampshire municipalities | State level, municipality setting | Food and nutrition, physical activity and breast-milk feeding (including food promotion, food retail, food provision and food prices) | The existence of implementation of policies and infrastructure supports | Quantitative method:  cross-sectional survey using online self-administered questionnaires with municipal representatives | The Implementation and Measurement Guide of Recommended Community Strategies and Measurements to Prevent Obesity in the United States  [Yes vs No per item] | Medium |
| Schwartz et al. (2012) [47] | USA (Connecticut) | To assess the strength and comprehensiveness of 1 state’s written district policies using a coding tool, and tested whether these traits predicted school-level implementation and practices | Federal level, school setting | Food and nutrition, physical activity and education (including food provision) | The level of implementation of district School Wellness policies | Quantitative method:  cross-sectional survey using self-administered questionnaires with school principals | The 96 items Wellness School Assessment Tool (WellSAT-96) [0, 1, 2 per item where 0=policy being not in place or don’t know and 2=policy being fully in place] | Medium |
| Phillips et al. (2012) [48] | USA (Arkansas) | To assess implementation of school policies related to nutrition and physical activity | State level, school setting | Food and nutrition, and physical activity (including food provision and food promotion) | The existence and level of implementation of nutrition and physical activity policies in Arkansas public schools from 2004 through 2009 | Quantitative method:  cross-sectional survey using self-administered questionnaires with school principals and district superintendents | Scoring policy index system tool [0, partial, 1 per indicator where 0=the policy did not exist or did not meet recommendations and 1=the policy existed and met recommendations] | Low |
| Budd et al. (2012) [49] | USA | To characterize the school wellness policy environment nationally and identify factors influencing the quality and effectiveness of policy implementation | Federal level, school setting | Food and nutrition, physical activity and education (including food provision) | The existence of implementation of the School Wellness policies | Quantitative method:  cross-sectional survey using self-administered questionnaires with high school representatives | The School Wellness Policies Implementation Questionnaire tool  [Yes, No, Not sure per indicator where Yes=the school took action to implement the policies, and No=the school did not take action to implement the policies] | Low |
| Beam et al. (2012) [50] | USA | To evaluate interim progress in schools receiving hands-on  training from the Healthy Schools Program, the nation’s largest school-based program aimed at preventing childhood  obesity | Federal level, school setting | Food and nutrition, physical activity and education (including food provision) | The existence of implementation of the Healthy Schools Program | Quantitative method:  cross-sectional survey using self-administered questionnaires with school representatives (e.g. school directors/principals teachers and food service managers) | The Healthy Schools Program tool  [Yes vs No per indicator and comparison to baseline] | Low |
| Pitt Barnes et al. (2011) [51] | USA | To describe the content of local wellness policies of 6 US school districts and steps taken toward their implementation and evaluation | Federal level, school setting | Food and nutrition, physical activity and education (including food provision) | The existence of implementation of Local School Wellness policies | Qualitative methods:  cross-sectional study using document review, school visits and in-depth interviews with key stakeholders (including  food service directors and physical education staff,  principals, teachers, parents, and community partners) | Interview questions based on information from document review  [narrative report] | Low |
| Gaines et al. (2011) [52] | USA (Alabama) | To evaluate wellness policies created by Alabama public school districts and progress made in the implementation of Alabama State Department of Education (ALSDE) school food and nutrition mandates | Federal level, school setting | Food and nutrition, physical activity and education (including food provision and food promotion) | The existence of implementation of Alabama public school district Wellness policies against federal requirements, and of districts’ implementation of Alabama State Department of Education mandates | Quantitative method:  cross-sectional survey using self-administered questionnaires with public school districts | The policy content checklist  [proportion of districts implemented (%)] | Low |
| Haire-Joshu et al. (2010) [53] | USA (Missouri) | To develop the Missouri  Obesity, Nutrition, and Activity Policy Database, a geographically representative baseline of Missouri’s existing obesity-related local policies on healthy eating and physical  activity | State level, various setting | Healthy eating and physical activity including food environments  (no specific details on food environments given) | The existence of implementation of obesity-related policies | Qualitative methods:  cross-sectional study using document review and interviews with government and non-government informants | Open-ended questions  [proportion of policies implemented by setting (%)] | Low |
| Belansky et al. (2010) [54] | USA (Colorado) | To describe changes  in evidence-based practices related to healthy food consumption before and after Local Wellness Policies (LWPs) implementation; contents of districts’ LWPs related to nutrition, including comprehensiveness and strength of LWP wording; and school foodservice managers’ impressions about the impact of the LWP on school cafeteria practices | Federal level, school setting | Food and nutrition, physical activity and education (including food provision) | The existence of implementation of Local School Wellness policies | Mixed methods:  cross-sectional study using in-depth interviews with foodservice managers and self-administered questionnaires with school principals, foodservice managers and teachers | The 96 items Wellness School Assessment Tool (WellSAT-96) [0, 1, 2 per item where 0=policy being not in place or don’t know, and 2=policy being fully in place]  (detail not given for interview) | Medium |
| Longley et al. (2009) [55] | USA | To examine the process and outcome of wellness policy development in school districts | Federal level, school setting | Food and nutrition, physical activity and education (including food provision) | The existence of implementation of district Wellness School policies before and after the federal mandate | Mixed methods:  cross-sectional study using telephone interviews and self-administered questionnaires with school foodservice directors | Open-ended questions for interviews  [narrative report]  (tool not specified for questionnaire)  [proportion of policies in place (%)] | Low |
| Leowski et al. (2009) [56] | 11 countries in South East Asia (SEA) region | To assess the status of national capacity for prevention and control of non-communicable diseases (NCDs) in the Member States of the South-East Asia (SEA) Region of the World Health Organization (WHO) | National level  (detail on setting not specified) | NCDs and risk factors including policies and programs recommended by the WHO Global Strategy on Diet, Physical Activity and Health  (including food promotion, food labelling, food prices and food composition) | The existence of implementation of national legislation, policies, strategies, and programmes on NCD prevention and control including the WHO Global Strategy on Diet, Physical Activity and Health | Quantitative method:  cross-sectional survey using self-administered questionnaires with the Ministries of Health  of the countries of the SEA Region | Adapted WHO global questionnaire tool on assessment of the national capacity for NCD prevention and control in 2001 [comparison to baseline] | Medium |
| Yeatman (2008) [57] | Australia | To determine the current level of activity of Australian local governments in twenty-nine food and nutrition action areas and whether the level of activity had changed between 1995 and 2007 | Local government level, rural and urban settings | Food and nutrition and education (including food retail and food provision) | The level of implementation of activity of local governments between 1995 and 2007 | Quantitative method:  cross-sectional survey using self-administered questionnaires with local governments | Tool developed based on the framework of Lester’s 1994 overview of the Australian food and nutrition system [Yes vs No per item/indicator] | Low |
| Probart et al. (2008) [58] | USA (Pennsylvania) | To assess local wellness policies established by Pennsylvania public school districts, compare these policies to local wellness policy mandate requirements, and provide information about local wellness policy development and implementation | Federal level, school setting | Food and nutrition, physical activity and education (including food provision) | The existence of implementation of Local School Wellness Policies | Quantitative method:  cross-sectional survey using self-administered questionnaires with school district representatives | The Local Wellness Policy Checklist  [Yes vs No per item/indicator] | Low |
| Action for Healthy Kids (2008) [59] | USA | To provide a snapshot of the state of school wellness after more than five years of work by Action for Healthy Kids, and others, at the national, state, and grassroots levels | Federal level, school setting | Food and nutrition, physical activity and education (including food provision and food composition) | The level of implementation of School Wellness policies after more than five years of work by Action for Healthy Kids, and others | Qualitative methods:  cross-sectional study using in-depth interviews with authorities on health and education, and school observations | Open-ended questions  [narrative report and proportion of respondents reporting implementation (%)] | N/A |
| Molaison et al. (2007) [60] | USA (Pennsylvania, Idaho, Arkansas, Mississippi) | To identify attitudes of school nutrition directors, principals, teachers, and parents regarding a Local Wellness Policies (LWP) and barriers related to implementation of a LWP | Federal level, school setting | Food and nutrition, physical activity and education (including food provision) | The existence of implementation of Local School Wellness policies | Mixed methods:  cross-sectional study using focus group interviews and self-administered survey questionnaires with principals, teachers, parents, school nutrition directors, and community professionals | Open-ended questions for focus group  [narrative report and frequency of comments on specific emerging issues]  (tool not specified for questionnaire)  [proportion of respondents reporting implementation (%)] | Low |
| Yee et al. (2006) [61] | USA | To assess the progress of the first 20 states funded by the Nutrition and Physical Activity Program to Prevent Obesity and Other Chronic Diseases | National level  (detail on setting not specified) | Nutrition and physical activity including food environments  (no specific details on food environments given) | The existence of implementation of the nutrition and physical activity program to prevent obesity and NCDs | Qualitative method:  cross-sectional study using document review | The 2004 state semi-annual reports for 21 funded states  [proportion of states reporting implementation (%)] | Low |
| Lang et al. (2006) [62] | Scotland | To examine the progress that has been made  in the implementation of the Scottish Diet Action Plan since 1996 – what has been achieved and what remains to be done | National and local levels, various settings | Food and nutrition, breastfeeding and health education (including food provision, food retail, food composition, food promotion, food labelling, food prices and food production) | The existence and level of implementation of the Scottish Diet Action Plan since 1996 | Mixed methods:  cross-sectional study using document review, expert reviews, self-administered questionnaires with government and non-government stakeholders, and discussion among key stakeholders | (tool not specified for questionnaire)  [narrative report]  List of key questions for discussion [narrative report] | N/A |
| Lower et al. (2004) [63] | Australia  (rural Western Australia) | To assess the implementation of the Australian core functions of public health in rural Western Australia | State level, local setting | NCDs and risk factor prevention, including promoting and supporting healthy lifestyle and healthy environments  (no specific details on food environments given) | The existence and level of implementation of the Australian public health core functions | Mixed methods:  cross-sectional study using self-administered questionnaires and semi-structured interviews with public health practitioners | Tool developed based on Australian core functions of public health as defined by the National Public Health Partnership [score 0-2 per item where 0=action is either rare or not undertaken at all, and 2=action was frequently part of current practice] | Low |
| Institute of Medicine (2013) [64] | USA | To measure progress in obesity prevention, including new metrics  for each goal area and strategy and  notes whether data for tracking change are available at the community and national levels | Federal, state, and community levels, various settings | Food and nutrition, physical activity and education (including food provision, food retail, food labelling and food promotion) | The existence of implementation of policies and programs towards proposed indicators | Guideline developed by the Institute of Medicine committee | Proposed 83 indicators in five goal areas | N/A |
| WHO (2008) [65] | Global | To measure the implementation of the  WHO Global Strategy on Diet, Physical Activity and Health (DPAS) at country level and proposes a framework and indicators for this purpose | National and sub-national levels, various settings | Diet and physical activity (including food labelling, food retail, food prices, food promotion, food provision and food composition) | The existence of implementation of policies and actions recommended in the WHO Global Strategy on Diet, Physical Activity and Health | Framework | Process, output and outcome indicators | N/A |
| Tagtow et al. (2011) [66] | USA (Iowa) | To measure the  health of Iowa’s food system through a report card leading to  recommendations for research, programs and policies to ensure a food system that supports healthier Iowans, communities, economies, and the environment | National level  (detail on setting not given) | Food system (including food marketing, food retail and food production) | The level of implementation of actions in Iowa’s food system | Quantitative method:  cross-sectional survey using self-administered questionnaires with key stakeholders | Iowa Food System Report Card  [data comparison over 10 years] | N/A |
| Fox et al. (2014) [67] | 10 countries from Asia and Latin America regions | To assess national political commitment and opportunities to advance food and nutrition policy reform, completed by knowledgeable representatives from 10 countries | National level  (details on settings not given) | Food and nutrition policy including food security  (no specific details on food environments given) | The political commitment to implement the policy | Quantitative method:  cross-sectional survey using self-administered questionnaires with government and non-government stakeholders | The Political Commitment and Opportunity Measurement-Rapid Assessment Tool  [Yes vs No per indicator] | Medium |
