# Supplementary material for: A review of methods and tools to assess the implementation of government policies to create healthy food environments for preventing obesity and diet-related non-communicable diseases
Source: Implement Sci. 2016 Feb 4;11:15. doi: 10.1186/s13012-016-0379-5 (PMC4743239; doi:10.1186/s13012-016-0379-5)
Supplement: Supplementary file 3 — Summary of identified studies evaluating policy implementation process. (DOC 136 kb) [file 13012_2016_379_MOESM3_ESM.doc]

**Additional file 3:** Summary of identified studies evaluating the government implementation of food environment policies and actions for preventing obesity and diet-related NCDs

| **Author** | **Study country** | **Objective of the study** | **Policy levels and settings** | **Policy areas** | **Aspects measured by the study** | **Design and methods** | **Tools**  **[scales used]** | **Overall quality of method/tool** |
| --- | --- | --- | --- | --- | --- | --- | --- | --- |
| Ardzejewska et al. (2013) [33] | Australia | To investigate the barriers and facilitators to, and the extent of the implementation of, the New South Wales (Australia) ‘Healthy School Canteen Strategy’ | State level, school setting | Food provision | Implementation barriers and facilitators of the New South Wales ‘Healthy School Canteen Strategy’ | Qualitative method:  cross-sectional study using semi-structured interviews with school principals or deputy principals and the canteen managers | Open-ended questions  [narrative report] | Low |
| Holthe et al. (2011) [35] | Norway | To examine how schools implemented the national guidelines for healthy school meals and the extent to which the degree of implementation was related to the organizational capacity of schools | National level, school setting | Food provision | Implementation barriers and facilitators for the national guidelines in Norwegian schools | Mixed methods:  cross-sectional study using document review, self-administered questionnaires, focus group interviews with teachers and students, and school observations | Interview guide and observations using observation form  [narrative report]  (tool not specified for questionnaire)  [Yes, Partial, No per element] | Low |
| Rodriguez-Fernandez et al. (2014) [36] | 53 WHO European Member States | To assess current salty reduction policies in countries of the WHO European Region against the backdrop of varying levels of human development adjusted for income, education and health (longevity) inequalities | National level  (details on settings not given) | Food composition and food labelling | Implementation barriers of salt reduction policy | Qualitative method:  cross-sectional study using document review and electronic communication with the WHO Nutrition Counterparts in the European Member States | The EU framework and eight essential steps proposed by WHO  [narrative report] | Medium |
| WHO/Europe (2013) [42] | Norway | To evaluate the Norwegian Action Plan on Nutrition 2007–2011 | National and local levels, various settings | Food and nutrition, physical activity and education (including food composition, food provision, food retail, food labelling, food prices and food promotion) | - Governance  - Implementation monitoring and evaluation  - Funding support  - Policy communication | Qualitative methods:  cross-sectional study using document review and in-depth interviews with policy makers and government and non-government stakeholders | Thematic matrix for guiding the interviews  [narrative report] | High |
| Mâsse et al. (2013) [43] | Canada | To explore the factors which impeded or facilitated the implementation of publicly mandated school-based physical activity and nutrition guidelines in the province of British Columbia (BC), Canada | Provincial level, school setting | Food and nutrition, physical activity and education (including food provision) | - Implementation barriers and facilitators of publicly mandated school-based guidelines  - Implementation styles (requiring all students to participate, or providing more opportunities to participate but not requiring) | Qualitative method:  cross-sectional study using semi-structured interviews with school principals and teachers/school informants | Open-ended questions  [narrative report] | Low |
| Barnidge et al. (2013) [45] | USA | To (1) identify types of environmental and policy interventions being implemented in rural communities to promote physical activity or healthy eating, (2) identify barriers to the implementation of environmental or policy interventions, and (3) identify strategies rural communities have employed to overcome these barriers | Federal level, community setting | Food and nutrition, physical activity and education  (including food provision and food production) | Implementation barriers and facilitators of environmental or policy interventions to promote physical activity and/or healthy eating in rural communities | Qualitative method:  cross-sectional study using in-depth interviews with public health professionals from non-profit or local governments | Interview protocol  [narrative report] | Low |
| Schwartz et al. (2012) [47] | USA  (Connecticut) | To assess the strength and comprehensiveness of 1 state’s written district policies using a coding tool, and tested whether these traits predicted school-level implementation and practices | Federal level, school setting | Food and nutrition, physical activity and education (including food provision) | Implementation barriers of School Wellness policies | Quantitative method:  cross-sectional survey using self-administered questionnaires with school principals | The WellSAT-96 tool [Yes vs No per item where Yes=the school experienced this barrier, and No=the school did not experience this barrier] | Medium |
| Budd et al. (2012) [49] | USA | To characterize the school wellness policy environment nationally and identify factors influencing the quality and effectiveness of policy implementation | Federal level, school setting | Food and nutrition, physical activity and education (including food provision) | Implementation barriers and facilitators of the School Wellness policies | Quantitative method:  cross-sectional survey using self-administered questionnaires with high school representatives | The School Wellness policies Implementation Questionnaire tool  [Yes, No, Not sure per indicator where Yes=this factor influenced the policy implementation, and No=this factor did not influence the policy implementation] | Low |
| Gaines et al. (2011) [52] | USA (Alabama) | To evaluate wellness policies created by Alabama public school districts and progress made in the implementation of Alabama State Department of Education (ALSDE) school food and nutrition mandates | Federal level, school setting | Food and nutrition, physical activity and education (including food provision and food promotion) | Barriers and facilitators that significantly impact policy compliance and implementation with district Wellness policies and the state mandates | Quantitative method:  cross-sectional survey using self-administered questionnaires with public school districts | A policy content checklist  [proportion of districts per component required for implementation (%)] | Low |
| Haire-Joshu et al. (2010) [53] | USA  (Missouri) | To develop the Missouri  Obesity, Nutrition, and Activity Policy Database, a geographically representative baseline of Missouri’s existing obesity-related local policies on healthy eating and physical  activity | State level, various settings | Healthy eating and physical activity including food environments  (no specific details on food environments given) | Funding | Qualitative methods:  cross-sectional study using document review and interviews with government and non-government informants | Open-ended questions  [proportion of states reporting funding availability and unavailability for policy implementation (%)] | Low |
| Longley et al. (2009) [55] | USA | To examine the process and outcome of wellness policy development in school districts | Federal level, school setting | Food and nutrition, physical activity and education (including food provision) | Implementation barriers and facilitators for district School Wellness policies before and after the federal mandate | Mixed methods:  cross-sectional study using telephone interviews and self-administered questionnaires with school foodservice directors | Open-ended questions for interviews  [narrative report],  (tool not specified for questionnaire) [proportion of respondents per indicator which available before and after the federal mandate (%)] | Low |
| Action for Healthy Kids (2008) [59] | USA | To provide a snapshot of the state of school wellness after more than five years of work by Action for Healthy Kids, and others, at the national, state, and grassroots levels | Federal level, school setting | Food and nutrition, physical activity and education (including food provision and food composition) | Implementation barriers and challenges of School Wellness policies after more than five years of work by Action for Healthy Kids, and others | Qualitative methods:  cross-sectional study using in-depth interviews with authorities on health and education, and school observations | Open-ended questions for interviews  [narrative report and proportion of respondents reporting each type of barriers and facilitators (%)] | Low |
| Molaison et al. (2007) [60] | USA  (Pennsylvania, Idaho, Arkansas, Mississippi) | To identify attitudes of school nutrition directors, principals, teachers, and parents regarding a Local Wellness Policies (LWP) and barriers related to implementation of a LWP | Federal level, school setting | Food and nutrition, physical activity and education (including food provision) | Barriers and benefits of implementing Local School Wellness Policies | Mixed methods:  cross-sectional study using focus group interviews and self-administered survey questionnaires with principals, teachers, parents, school nutrition directors, and community professionals | Open-ended questions for focus group  [narrative report and frequency of comments on specific emerging issues]  (tool not specified for questionnaire)  [proportion of respondents per indicator (%)] | Medium |
| Lang et al. (2006) [62] | Scotland | To examine the progress that has been made in the implementation of the Scottish Diet Action Plan since 1996 – what has been achieved and what remains to be done | National and local levels, various settings | Food and nutrition, breastfeeding and health education (including food provision, food retail, food composition, food promotion, food labelling, food prices and food production) | Barriers and opportunities of implementing and achieving Scottish Diet Action Plan targets | Mixed methods:  cross-sectional study using document review, reviews by international experts, and self-administered questionnaires with government and non-government stakeholders | (tool not specified for questionnaire)  [narrative report]  List of key questions for discussion [narrative report] | N/A |
| Sanchez et al. (2014) [68] | USA  (New Mexico) | To examine school nutrition and physical activity policy implementation in two school districts in a northern New Mexico town | Federal level, school setting | Food and nutrition, physical activity and education (including food provision) | Implementation barriers and facilitators of School Wellness policies | Qualitative methods:  cross-sectional study using individual Interviews with district-level and school-level administrators, and focus group interviews with students | Interview and focus group guides [narrative report] | Low |
| Middleton et al. (2014) [69] | UK | To explore the experiences of  ‘implementation’ by stakeholders of a large community-based obesity prevention programme, facilitated by a National Health Service Care Trust in the north-east of England, UK | Local level, various settings | Food and nutrition, and physical activity  (no specific details on food environments given) | Implementation barriers and facilitators of obesity prevention programme | Qualitative methods:  cross-sectional study using semi-structured interviews with local authority and Care Trust workers at strategic and commissioning level and local people, and focus group interviews with local authority and Care Trust workers responsible for coordination, administration and delivery | Open-ended questions, small prompts, probes and follow-up questions [narrative report] | Low |
| Fagen et al. 2014) [70] | USA  (Chicago) | To evaluate implementation of Communities Putting  Prevention to Work (CPPW) initiative in suburban Cook County | County level, school setting | Food and nutrition, physical activity and education (including food provision and food retail) | Implementation barriers and facilitators of Policy, Systems, and Environmental change strategy | Qualitative method:  cross-sectional study using in-depth interviews with school district personnel | Open-ended interview questions  [narrative report] | Low |
| Taylor et al. (2010) [71] | Canada | To 1) provide an overview of key issues in monitoring and evaluating school nutrition and  physical activity policies in Canada and 2) identify areas for further research needed to strengthen the evidence base and inform the development of effective approaches to monitoring and evaluation | National level, school setting | Food and nutrition and physical activity according to recommendations of the WHO Global Strategy on Diet, Physical Activity and Health  (including food promotion, food labelling, food prices and food composition) | - Partnerships  - Infrastructure supports | Qualitative method:  cross-sectional study using document review | The WHO framework to monitor and evaluate implementation of the Global Strategy on Diet, Physical Activity and Health  [narrative report] | N/A |
| MacLellan et al. (2010) [72] | Canada | To explore parent and student perceptions of barriers and facilitating factors influencing the implementation of school nutrition policies | District level, school setting | Food and nutrition and education (including food provision) | Implementation barriers and facilitators of school nutrition policies | Qualitative methods:  cross-sectional study using focus group interviews with students and in-depth interviews with their parents | Interview guide  [narrative report] | Low |
| Harris et al. (2010) [73] | USA  (West Virginia) | To describe the processes and methods used to evaluate efforts to implement the legislation | State level, school setting | Food and nutrition, physical activity and education (including food provision) | Implementation barriers and facilitators of the West Virginia Healthy Lifestyles Act | Mixed methods:  cross-sectional study using self-administered questionnaires with county superintendents, school principals, school nurses and physical education teacher, and in-depth interviews with state legislators, superintendents, principals, physical education teachers, school nurses, parents of school-aged children and students | (tool not specified for questionnaire)  [proportion of respondents per indicator (%)]  Semi-structured questions  [narrative report] | Low |
| Agron et al. (2010) [74] | USA | To understand the wellness  environment in school districts across the country and to identify challenges districts  face and needs they have in order to effectively implement, monitor, and evaluate school  wellness policies | Federal level, school setting | Food and nutrition, physical activity and education (including food provision) | Implementation barriers and facilitators of School Wellness policies | Qualitative methods:  cross-sectional study using focus group interviews with school board members and policy/government services directors, and in-depth phone interviews with stakeholders from school districts and a state-level collaboration implementing the policies | Discussion guide  [narrative report] | Low |
| Allender et al. (2009) [75] | Australia | To investigate barriers and facilitators to local government  policy change in relation to environments for healthy eating and physical activity | Local level  (detail on setting not given) | Food and nutrition and physical activity  (no specific details on food environments given) | Implementation of barriers and facilitators of policies for obesity prevention | Qualitative method:  cross-sectional study using semi-structured interviews with representatives of local government | Interview guide  [narrative report] | Low |
| McDonnell et al. (2006) [76] | USA  (Pennsylvania) | To examine the role of school foodservice directors in  development and enforcement of local school wellness policies, their feelings about these roles, and perceived barriers | Federal level, school setting | Food and nutrition, physical activity, education (including food provision) | Implementation barriers of Local School Wellness policies | Qualitative method:  cross-sectional study using focus group interviews with school foodservice directors | Standard recommended focus group protocols developed by Krueger & Casey (2000)  [narrative report] | Low |
| Dunět et al. (2005) [77] | USA  (nine states) | To create and test the State Plan Index and use it to evaluate the quality of nine state plans aimed  at preventing and reducing obesity | State level  (detail on setting not given) | Food and nutrition, physical activity, breastfeeding and education (including food provision, food retail, and food labelling) | - Stakeholder engagement - Resources for policy implementation - Evaluation mechanism | Quantitative method:  cross-sectional survey using self-administered questionnaires with state staff | The State Policy Index Tool [0-5 per index where 0= was not mentioned or did not exist, and 5=high quality (item exists and is perceived as being often close to ideal] | N/A |
| Abery et al. (2014) [78] | Australia | To discuss the implementation  of the ‘Right Bite Healthy Food and Drinks Strategy for South Australian Schools and Preschools’ (Right Bite) | State level, school setting | Food provision | Implementation barriers of mandatory nutrition guidelines for school canteens | Qualitative methods:  cross-sectional study using semi-structured interviews with school principals, canteen managers and parents, focus group interviews with students, and participant observation through canteen | Open-ended interview questions  [narrative report] | Low |
| Crammond et al. (2013) [79] | Australia | To investigate barriers and  facilitators to implementing regulatory interventions to prevent obesity within the  executive arm of the Australian Commonwealth Government | National level  (detail on setting not given) | Food and nutrition, food safety  (including food provision, food promotion, food labelling, food production, food prices and food retails) | Implementation barriers and facilitators of regulatory interventions to prevent obesity | Qualitative methods:  cross-sectional study using document review and semi-structured interviews with managerial-level government officers | Semi-structured questions  [narrative report] | Medium |
| Shill et al. (2012) [80] | Australia | To identify regulatory interventions targeting the food environment, and barriers/facilitators to their implementation at the Australian state government level | State level, various settings | Food promotion, food provision, food production, food prices and food retails | Implementation barriers and facilitators of regulatory interventions targeting food environments | Qualitative method:  cross-sectional survey using in-depth interviews with senior representatives from state/territory governments, statutory authorities and non-government organizations | Semi-structured questions  [narrative report] | Medium |
| Chung et al. (2012) [81] | Australia | To understand the perceptions of senior representatives from  Australian state and territory governments, statutory authorities and non-government organisations regarding the  feasibility of state-level government regulation of television marketing of unhealthy food to children in Australia | State level  (detail on setting not given) | Food promotion | Implementation barriers and facilitators of regulation of television marketing of unhealthy food to children | Qualitative method:  cross-sectional study using in-depth semi-structured interviews with senior representatives from state and territory government departments, statutory authorities and non-government organisations | Semi-structured questions  [narrative report] | Low |
